# The Effect of Thermocycling and Mechanical Loading on the Fracture Resistance of Graphene and All-Ceramic Anterior Crowns

**DOI:** 10.7759/cureus.61097

**Published:** 2024-05-26

**Authors:** Karri Akhil Sai Reddy, B Rajendra Prasad, Reddy Priya Darshini, Sai Ram Challa, Mondreti Naga Charishma, Sangani Sowjanya, Kakarlapudi Akhila Devi, Sai Swetha Turlapati, Pathuri Raveen Teja

**Affiliations:** 1 Department of Prosthodontics, Crown & Bridge and Implantology, Ganni Subba Lakshmi (GSL) Dental College & Hospital, Rajahmundry, IND

**Keywords:** graphene, mechanical loading, thermocycling, fracture resistance, ips e-max

## Abstract

Introduction

Fixed prosthodontic treatment involves the replacement of missing tooth structures with a variety of materials. Several newer metal-free ceramics have been developed in recent years to meet patients' aesthetic needs. The long-term performance of all ceramics, however, is unknown, necessitating a continuous evaluation of the materials' strength.

Aim

The aim of this study was to compare and evaluate the fracture resistance of IPS E max pressable crowns and graphene crowns, which are luted with Rely X U200 self-adhesive resin cement on the respective dies, as well as thermocycling of IPS E max pressable crowns and thermocycling of graphene crowns. The current review was conducted as an in vitro examination at the Division of Prosthodontics, GSL Dental School, Rajahmundry, Andhra Pradesh, India.

Materials and methods

On a typodont tooth, a shoulder finish line design was prepared and incisal reduction was performed. The tooth was scanned, designed, and milled to produce 18 metal dies made of cobalt-chrome alloy. These metal dies produced a total of (n=36) all-ceramic crowns, which were divided into two groups based on crown type: 18 IPS E max crowns and 18 graphene crowns. The participants were once again divided into two subgroups within each group, with the purpose of assessing fracture resistance. This evaluation was conducted using a universal testing machine both before and after subjecting the specimens to thermocycling. The obtained data were sent for statistical analysis.

Results

Fracture resistance values were reduced after thermocycling of both IPS E max and Graphene crowns. Without thermocycling, the fracture resistance values of IPS E max crowns were higher than those of graphene crowns.

Conclusions

The fracture resistance of IPS E max crowns exhibited a statistically significant increase when compared to graphene crowns. Additionally, it was shown that the fracture resistance of both materials was reduced upon exposure to thermocycling.

## Introduction

Dentistry is evolving at an exponential rate both scientifically and artistically, as it has close associations with the fields of material science, chemistry, engineering, and computer science. Restorative and prosthetic dentistry is widely recognized as a rapidly growing field within the subject of dentistry, Metal-free restorations became more popular as dental technology improved and aesthetic demands increased [1,2].

Fixed partial dentures (FPDs) that use a metal framework and porcelain face have demonstrated notable fracture resistance and have been extensively employed in clinical settings. However, these FPDs are not without their limitations, which include the visibility of the metal framework and the potential for gingival margin discoloration resulting from metal corrosion [3].

To address these downsides, all-artistic rebuilding efforts are viewed as the essential inclination for accomplishing extraordinarily appealing results. Numerous all-ceramic systems are currently being developed to fulfill the demanding criteria of restorative dentistry. The IPS E max press and IPS E max computer-aided design (CAD) frameworks use lithium disilicate as their essential material and are presented in both squeezed and computer-aided design, computer-aided manufacturing (CAM) milling, the IPS E max press is composed of ingots that derive their strength from the presence of finely scattered crystals inside a glassy matrix, eliminating the need for an additional crystallization process [4-6].

Recently, novel allotropes of carbon, such as graphene, came into existence. Graphene is a novel 2D carbon nanoform. Because of its unique properties, the sp2-bonded carbon atom has garnered a lot of attention with remarkable physical and chemical properties and offers good esthetics. Graphene and its derivates have shown outstanding potential in many fields such as composite materials, nanoelectronics, sensors, water purification, biomedical applications, biosensing, tissue engineering, and dentistry. Among its principal properties are high electrical conductivity, high traction resistance, low density, and low coefficient of thermal expansion.

As the oral cavity is dynamic in nature, temperature and loading conditions put restorative materials in jeopardy; it is critical to simulate those circumstances in vitro. Thermomechanical fatigue loading is used to replicate clinical circumstances [7,8].

Hence, the present study sought to assess the material's resistance to fracture. Ceramic IPS E max press and graphene crowns undergo thermocycling before and after being heat-pressed. These crowns are then bonded with Rely X U200 self-adhesive resin (3M ESPE, Seefeld, Germany).

## Materials and methods

Preparation of typodont teeth

The maxillary central incisors (typodont teeth) were prepared according to the following criteria: a 6-degree convergence angle, a 1.2-mm labial shoulder finish line, a 1-mm lingual shoulder finish line, and a 2-mm incisal reduction. To lessen the concentration of stress, all sharp line angles were rounded and smoothed.

Fabrication of metal dies

**Figure 1:**
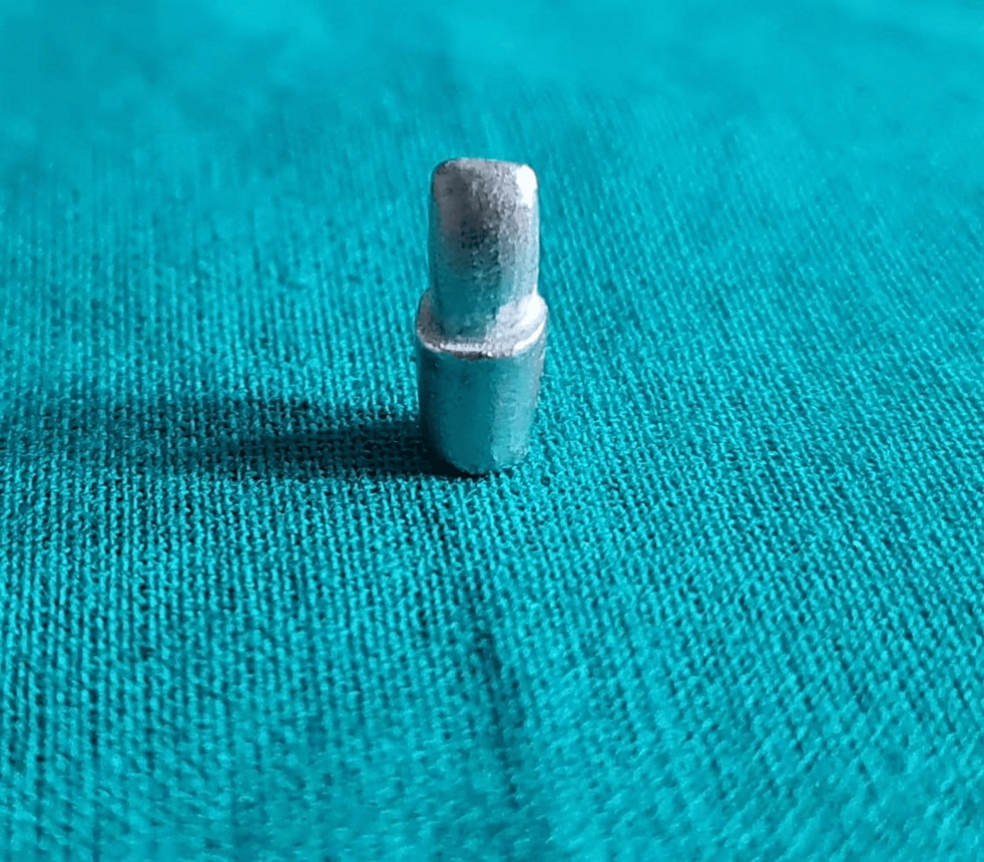
Image depicting the fabricated metal die using Co-Cr alloy (LaserForm)

The design was executed using Exocad. Upon completion of the calculation of the scanned image of the die, the subsequent step was the initiation of finish line tracing, which was then followed by the determination of the insertion axis. Once the final design was approved, it was subsequently transferred to the milling machine and fabricated using the DMP Dental 100 system. This system is recognized for its direct metal printing capabilities, enabling the production of a superior metal die. The die was generated using LaserForm Co-Cr (B) material (3D Systems, Rockhill, SC) within a 3D framework (Figure 1).

Grouping of the samples

**Figure 2:**
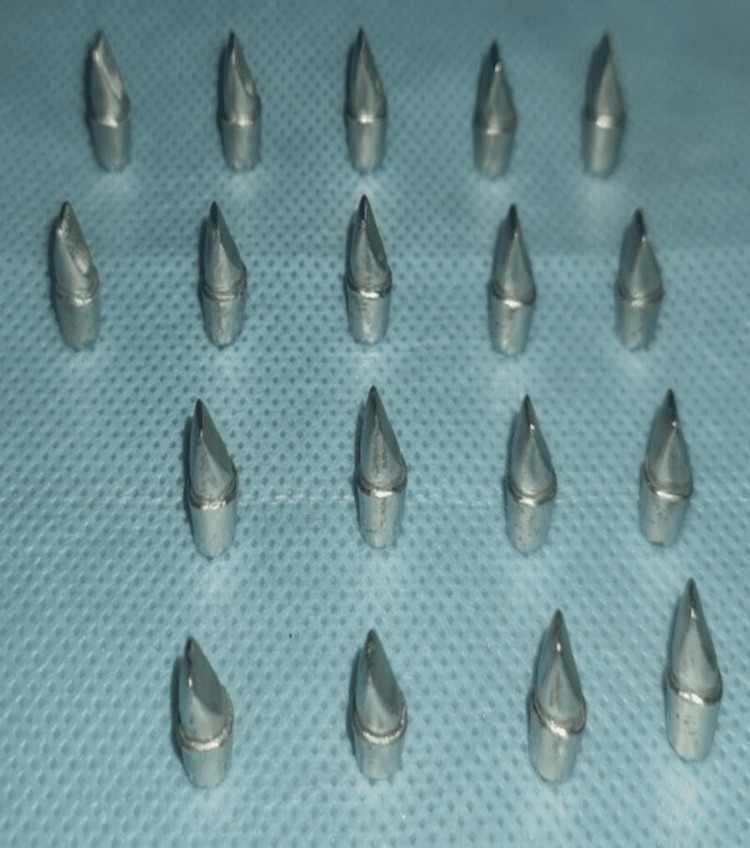
Image depicting the total 18 metal dies

A total of 18 metal dies, which were fabricated, were taken. A total of 36 ceramic crowns (IPS E max and graphene) were fabricated with each metal die, and the constructed specimens were split into two major groups, 1 and 2, each with 18 crown specimens (Figure 2):

Group 1: 18 IPS E max press all-ceramic crowns; Group 2: 18 graphene crowns

Group 1 contained 18 pressable crowns and was divided equally into two subgroups: 1 (a) and 1 (b), with nine samples in each subgroup.

Subgroup 1 (a): To evaluate the strength against fracture in the absence of thermocycling, it is necessary to conduct an analysis.

Subgroup 1 (b): To assess the fracture resistance after thermocycling.

Group 2 contained graphene crowns and was further divided equally into two subgroups: 2 (a) and 2 (b), with nine samples in each subgroup.

Subgroup 2 (a): To assess the fracture resistance in the absence of thermocycling, it is necessary to conduct an evaluation.

Subgroup 2 (b): To evaluate the strength against fracture.

Fabrication of graphene crowns

These designed copings were nesting using CAM software in the hyperDENT® Classic (Follow-Me, Munich, Germany). In the CAD/CAM milling machine (ARUM), the G-CAM discs were placed, and milling of the graphene crowns with standard measurements was prepared.

Fabrication of IPS E max crowns

A DOF (EDGE) scanner was used to scan metal dies, and coping was designed using hyperDENT (Classic) CAM software. Wax patterns were digitally fabricated from the wax blocks. Investing was performed in the furnace at 800 degrees Celsius, and a muffle furnace was used for the burnout. Mold space was created, and IPS E max press ingots were pressed using a pressing furnace to form E max copings.

Cementation of crowns to the metal dies

**Figure 3:**
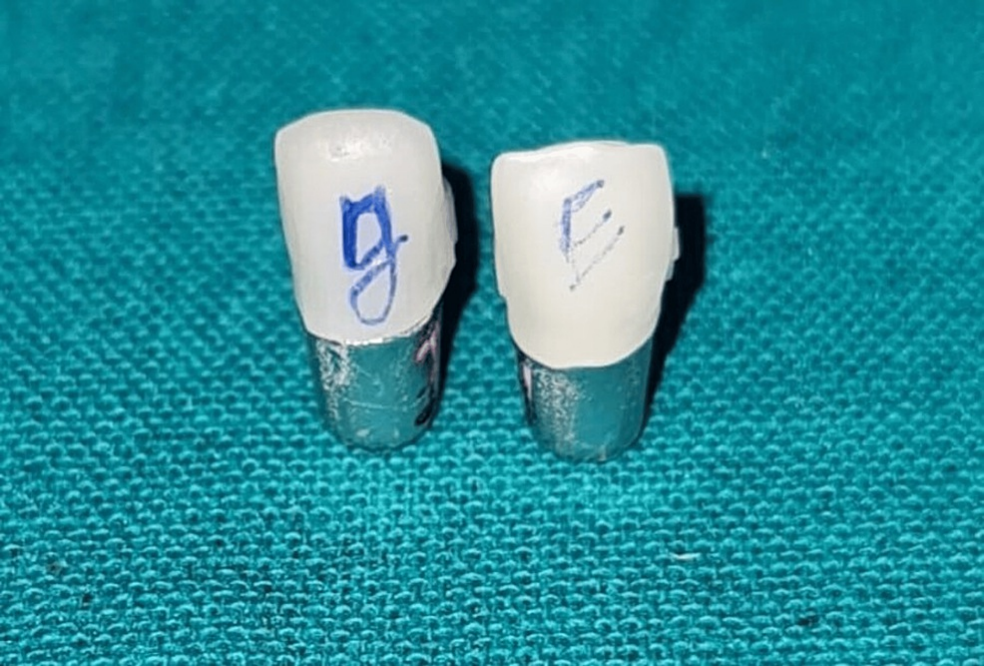
Image depicting the crowns luted to their respective dies.

The IPS E max and graphene crowns underwent a 20-second etching process using hydrofluoric acid, followed by a comprehensive rinsing and air-drying procedure. Subsequently, the silane coupling agent was administered and subjected to a drying process lasting 60 seconds (Figure 3).

**Figure 4:**
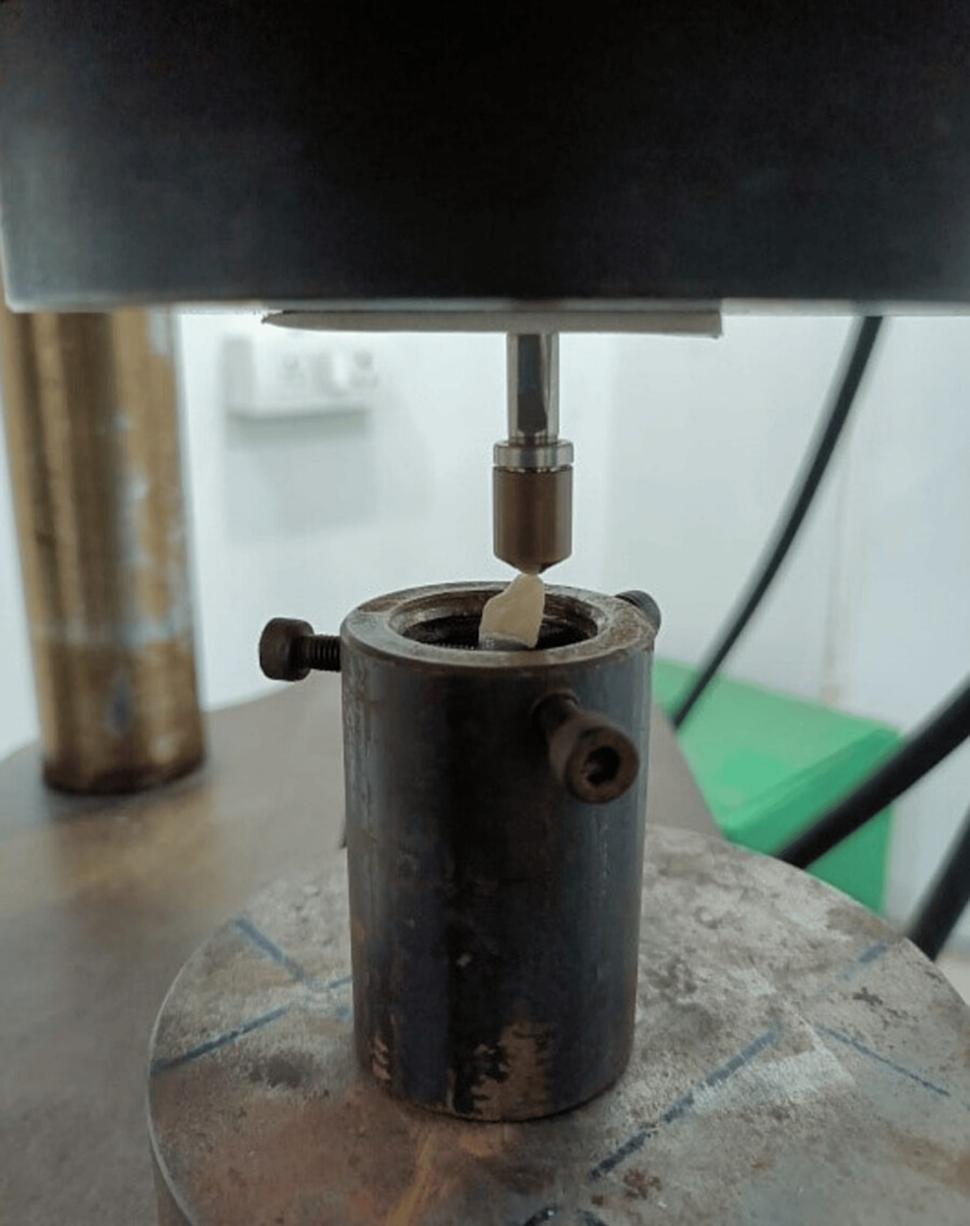
Image illustrating the process of fracture resistance testing using a universal testing machine.

The crown fitting surfaces were fabricated using a self-adhesive resin cement known as Rely X U200. Each restoration within the complete set was seated for a duration of 5 minutes, employing finger pressure specifically applied to its corresponding metal abutments. The surplus material was eliminated through the use of a scaler. Conforming to the guidelines provided by the manufacturer, the crowns underwent thermocycling, followed by an evaluation of their fracture resistance using a universal testing machine (Figure 4).

Thermocycling procedure

The samples were submerged in distilled water at a temperature of 37 degrees Celsius for one day before keeping in a thermocycler. Subsequently, the samples underwent 1,500 cycles in a water bath with temperatures ranging from 5 to 55 degrees Celsius. Each bathing session had a duration of 30 seconds.

Fracture resistance testing

The entirety of the specimens was securely fastened and inserted into a universal testing apparatus. When the crosshead velocity is set to 0.5mm/min, the ball is permitted to undergo vertical movement in a compressive mode at an angle of 135 degrees relative to the long axis of the crown. For each sample, the peak-load fracture was measured in Newtons (N) and tabulated.

## Results

Table 1 presents the descriptive statistics for fracture resistance (N) of IPS E max and graphene crowns without and after thermocycling. The fracture resistance values for IPS E max crowns without thermocycling ranged between 1,280 N and 2,580 N, with a mean of 1,760±392.42 N. After thermocycling, the fracture resistance values for IPS E max crowns ranged between 1,260 N and 2,040 N, with a mean of 1,632.22±292.56 N. For graphene crowns without thermocycling, the fracture resistance values ranged between 520 N and 940 N, with a mean of 706.67±177.2 N; after thermocycling, the mean was 613.33±86.6 N.

**Table 1:** Descriptive statistics for fracture resistance (N) of IPS E max and graphene crowns without and after TC TC, thermocycling

Crowns	N	Range	Minimum	Maximum	Mean	Standard Deviation
IPS E max without TC	9	1,300	1,280	2,580	1,760.00	392.428
IPS E max after TC	9	780	1,260	2,040	1,632.22	292.565
Graphene without TC	9	420	520	940	706.67	177.200
Graphene after TC	9	260	460	720	613.33	86.603

Table 2 shows the comparison of fracture resistance (N) of IPS E max crowns without and after thermocycling. The fracture resistance of the IPS E max crowns was found to be reduced after thermocycling. However, this difference between fracture resistance values without and after thermocycling was not statistically significant for the IPS E max crowns.

**Table 2:** Comparison of fracture resistance (N) of IPS E max crowns without and after TC Wilcoxon signed rank test was used for comparison; p≤0.05 was considered statistically significant TC, thermocycling

Crowns	N	Mean	Standard Deviation	Z-value	P-value
IPS E max without TC	9	1760.00	392.428	-0.771	0.44
IPS E max after TC	9	1632.22	292.565		

Table 3 shows the comparison of fracture resistance (N) of graphene crowns without and after thermocycling. The fracture resistance of the graphene crowns was reduced after thermocycling. However, this difference between fracture resistance values without and after thermocycling was not statistically significant for the graphene crowns.

**Table 3:** Comparison of fracture resistance (N) of graphene crowns without and after TC Wilcoxon signed rank test was used for comparison; p≤0.05 was considered statistically significant TC, thermocycling

Crowns	N	Mean	Standard Deviation	Z-value	P-value
Graphene without TC	9	706.67	177.200	-1.24	0.213
Graphene after TC	9	613.33	86.603		

Table 4 shows the comparison of fracture resistance (N) of IPS E max and graphene crowns without thermocycling. When compared to the IPS E max crowns, the fracture resistance values for the graphene crowns were significantly lower.

**Table 4:** Comparison of fracture resistance (N) of IPS E max and graphene crowns without TC Mann-Whitney U test was used for comparison; p≤0.05 was considered statistically significant. *Statistical significance TC, thermocycling

Group	N	Mean	Standard Deviation	Standard Error of the Mean	Z-value	P-value
IPS E max	9	1,760.00	392.428	130.809	-3.582	<0.001*
Graphene	9	706.67	177.200	59.067		

Table 5 shows the comparison of fracture resistance (N) of IPS E max and graphene crowns after thermocycling. When compared to the IPS Emax crowns, the fracture resistance values for the graphene crowns were significantly lower even after thermocycling.

**Table 5:** Comparison of fracture resistance (N) of IPS E max and graphene crowns after TC Mann-Whitney U test was used for comparison; p≤0.05 was considered statistically significant *Statistical significance TC, thermocycling

Group	N	Mean	Standard Deviation	Standard Error of the Mean	Z-value	P-value
IPS E max	9	1632.22	292.565	97.522	-3.578	<0.001*
Graphene	9	613.33	86.603	28.868		

Table 6 shows the overall comparison revealed a statistically significant difference between IPS E max and graphene crowns, with the IPS E max crowns having higher mean values.

**Table 6:** Overall comparison of fracture resistance (N) of IPS E max and graphene crowns Mann-Whitney U test was used for comparison; p≤0.05 was considered statistically significant. *Statistical significance TC, thermocycling

Group	N	Mean	Standard Deviation	Standard Error of the Mean	Z-value	P-value
IPS E max	18	1696.11	342.158	80.647	-5.13	<0.001*
Graphene	18	660.00	143.568	33.839		

## Discussion

The field of fixed prosthodontics has experienced a significant transition in recent times due to the advent of advancements in adhesive dentistry and ceramic technology. Dental ceramic technology is one of the most rapidly increasing branches of the scientific study of dental materials. Ceramic dental materials were an appealing alternative for patients and dentists because of their great aesthetic capability and biocompatibility [9,10].

The high strength necessary for all-ceramic restorations was obtained by developing ceramic materials with a much more crystalline phase. While the upgraded translucent stage adds to the improved strength and break opposition of pottery, it is additionally connected with elevated murkiness, possibly compromising the stylish allure of the last rebuilding [11].

Dental pottery can be sorted into three particular classes, as indicated by their translucent creation. This vitreous ceramic is predominantly composed of feldspar minerals, consisting mainly of silicon dioxide (silica or quartz) with a minimal crystalline structure. It is so far the best option to mimic the optical characteristics of the natural tooth structure; however, the application of monolithic restorations is limited due to their inferior mechanical properties [12,13].

Filler particles consisting of glass with a crystalline structure or a higher melting temperature in the base glass composition are used as a means to enhance the mechanical qualities [14]. The physical and mechanical properties are essentially impacted by the circulation, size, number, and type of the translucent stages. This characterization envelops a scope of contemporary earthenware frameworks described by varying degrees of translucent organization, including leucite glass-fired ceramics, lithium disilicate glass-clay, and glass-penetrated alumina-based pottery.

Due to the continuous replacement of the glassy phase with the crystalline phase to enhance the strength and durability of repairs, monophase ceramics such as alumina or zirconia exhibit the absence of a glassy matrix. One notable drawback associated with the enhanced ceramics is their increased opacity in comparison to glass ceramics. Typically, these materials are employed as foundational substrates that are overlaid with glassy ceramics to provide visually pleasing outcomes.

A diverse array of ceramic systems with different crystalline compositions has been developed through the use of manufacturing and processing processes, including alumina, zirconia, and lithium disilicate restorations. Alumina-based restorations have been widely used as a favored prosthodontic choice for an extended period of time. In light of the notable achievements observed in alumina-based ceramics, particularly the In-Ceram series developed by VITA Zahnfabrik (Bad Säckingen, Germany). The specific definition of the system's application has likely been refined due to the intricate and time-intensive nature of the procedure. This complexity poses challenges in attaining an accurate fit and may lead to internal faults that compromise the material's strength as a result of partial glass penetration [15].

Lithium disilicate glass-ceramic represents an additional viable option for all-ceramic restorations in terms of its competitive properties. Lithium disilicate as a restorative dental material under the brand name IPS Empress II was introduced in 1998 by Ivoclar Vivadent, a company based in Schaan, Liechtenstein. The current methodology uses a blend of the lost-wax technique and hot-squeezing process for clay ingots. A definitive microstructure is made out of an extraordinary design of thickly interconnected lithium disilicate gems. Extraneous compressive burdens emerge near the gems as a result of the divergent warm extension coefficients showed by lithium disilicate precious stones and the polished framework. These anxieties are accepted to upgrade the material's solidarity and advance break avoidance [16].

Machinable lithium disilicate blocks, specifically IPS E max CAD type, have been intended to be viable with PC-supported plan and PC-supported fabricating (CAD/CAM) innovation. The crystallization of these blocks happens in two unmistakable advances. During the underlying stage, the development of lithium-metasilicate precious stones at a centralization of 40 vol% prompts a flexural strength going from 130 MPa to 150 MPa; this composition permits easier machining and intraoral occlusal adjustment. During the last stage of crystallization, the glass matrix exhibits a crystal volume occupancy of 70%, resulting in an augmented resistance in the finished product [17].

During this particular phase, the pre-crystallized block undergoes a transformation, in which its original blue hue is altered to match the desired shade of the tooth. Lithium disilicate glass ceramics have exhibited notable levels of durability. The IPS Empress 2 crowns had a survival rate ranging from 95% to 100% after a period of five years and a survival rate of 95.5% after a duration of 10 years. After 4.6 years, Reich and Schierz reported a 96.3% survival rate for chairside-generated E max CAD crowns [18].

Shamseddine et al. found that milled wax patterns offered a better fit than the traditional method when comparing the fit of ceramic crowns made with versus without milled wax patterns. For the fit of pressed crowns, Shamseddine et al. contrasted additive and subtractive CAD-CAM wax pattern processing. Between the two CAD-CAM manufacturing processes, no statistically significant differences were found [19].

In a study by Rosentritt et al., lithium disilicate FDP samples were tested using a variety of fatigue testing protocols, including the magnitude of load applied, antagonist material, abutment material, loading frequency, loading condition (wet at constant temperature or thermocycling), mouth opening distance, periodontal ligament simulation, lateral movement, and aging device [20].

It was shown that each of these characteristics could exert a substantial influence on the outcomes of the tests. The fracture load following fatigue for a given number of cycles exhibited a range of values, spanning from 410 N to 1,713 N, contingent upon the loading parameters employed across distinct groups.

Thermocycling is a technique that can be employed to replicate the conditions found within the mouth cavity. Consequently, it is used in the simulation of the process of ageing. There is significant variation in the number of cycles and temperature extremes seen across different studies. The International Organization for Standardization (ISO) has created a standardized methodology to facilitate the interpretation and comparison of thermocycling test outcomes by investigators. According to this technique, a suitable artificial ageing test involves subjecting the material to 500 cycles in water within the temperature range of 5 to 55 degrees Celsius.

The samples went through a thermocycling interaction comprising of 1,500 cycles, with water showers set at temperatures of 5 to 55 degrees Celsius. Each shower has a dwell time of 30 seconds in each bath. Preceding the thermocycling, the samples were put away in refined water at a temperature of 37 degrees Celsius for 24 hours.

Limitations

This study could be limited due to the elastic modulus of the supporting die material. Metal dies exhibit less deformation than dentin because they are more rigid and possess a higher elastic modulus, which results in less shear stress at the inner crown surface; as a result, the obtained loads may be greater than those of dentin dies.

Furthermore, the mode of fracture failures and the weight applied to ceramic crowns during fracture testing were static and did not mimic dynamic clinical loading situations. More in vivo research is recommended to better understand the impact of the complex oral environment on the mechanical properties of various ceramic restorations, despite standardization challenges.

Thermocycling does not completely replicate the real clinical condition. To fully replicate the oral environment, we may increase thermocycling cycles to more than 1,500. We can also check the color of aesthetic restoration and its color stability for the long-term success of lithium disilicate and graphene crowns.

## Conclusions

There were no statistically significant changes in IPS E max before and after thermocycling in terms of fracture resistance, and there was no discernible change in the fracture resistance of graphene crowns before and after thermocycling and without thermocycling. IPS E max and graphene crowns differ significantly. In general, there was a statistically significant difference between the IPS E max and Graphene crowns, with IPS E max crowns having higher mean values regardless of the thermocycling method.

Following thermocycling, there were significant differences between the IPS E max and graphene crowns, with IPS E max crowns having higher mean values regardless of the thermocycling method.
